# Impact of Genetic Notification on Smoking Cessation: Systematic Review and Pooled-Analysis

**DOI:** 10.1371/journal.pone.0040230

**Published:** 2012-07-11

**Authors:** Sylviane de Viron, Johan Van der Heyden, Elena Ambrosino, Marc Arbyn, Angela Brand, Herman Van Oyen

**Affiliations:** 1 Operational Direction Public Health and Surveillance, Scientific Institute of Public Health, Brussels, Belgium; 2 Institute for Public Health Genomics (IPHG), Maastricht University, Maastricht, The Netherlands; 3 Research Institute CAPHRI - School for Public Health and Primary Care, Maastricht University, Maastricht, The Netherlands; 4 Research Institute GROW - School for Oncology and Developmental Biology, Maastricht University, Maastricht, The Netherlands; Sapienza University of Rome, Italy

## Abstract

**Objectives:**

This study aimed to evaluate the impact of genetic notification of smoking-related disease risk on smoking cessation in the general population. Secondary objectives were to assess the impact of genetic notification on intention-to-quit smoking and on emotional outcomes as well as the understanding and the recall of this notification.

**Methods:**

A systematic review of articles from inception to August 2011 without language restriction was realized using PubMed, Embase, Scopus, Web of Science, PsycINFO and Toxnet. Other publications were identified using hand search. The pooled-analysis included only randomized trials. Comparison groups were (i) high and low genetic risk versus control, and (ii) high versus low genetic risk. For the pooled-analysis random effect models were applied and sensitivity analyses were conducted.

**Results:**

Eight papers from seven different studies met the inclusion criteria of the review. High genetic risk notification was associated with short-term increased depression and anxiety. Four randomized studies were included in the pooled-analysis, which revealed a significant impact of genetic notification on smoking cessation in comparison to controls (clinical risk notification or no intervention) in short term follow-up less than 6 months (RR = 1.55, 95% CI 1.09–2.21).

**Conclusions:**

In short term follow-up, genetic notification increased smoking cessation in comparison to control interventions. However, there is no evidence of long term effect (up to 12 month) on smoking cessation. Further research is needed to assess more in depth how genetic notification of smoking-related disease could contribute to smoking cessation.

## Introduction

Smoking is a major public health problem worldwide and the most preventable cause of mortality and morbidity. It increases the risk of many diseases such as lung cancer, chronic obstructive pulmonary diseases and cardiovascular diseases [1], but smoking cessation higly contributes to the prevention of most of these harms. Every year, around 40% of smokers attempt to quit smoking for at least one day, but only few of them succeed: approximately 2% without any help and 20% with an adequate treatment [2], [3]. This highlights the importance of improving evidence-based interventions for smoking cessation, which could be enhanced by genetic notification of smoking-related disease risk. The goal of genetic notification is to allow smokers to adapt their behavior regarding their personal risks [4], [5].

Common diseases are highly dependent on multiple environmental and genomic factors. Both the cigarette consumption and the allele frequencies vary substantially between populations. Multiple single nucleotide polymorphisms (SNPs) are needed for the assessment of each specific smoking-related disease risk. Generally speaking, testing multiple SNPs for diverse smoking-related disease risks will identify smokers to be at higher risk of at least one disease. Different genes seem to be of interest in cancer risk prediction, among them: GSTM1, GSTT1, CYP2D6, L-myc, NQO1, and CYP1A1 [6]–[10].

Risk communication and health literacy are complex issues dealing with the use, the understanding and the recalling of a notification by the patient [4], [11]. Combination of a numeric, verbal and pictorial approach maximizes the understanding of the genetic risk [12]. Different models, such as the extended parallel process model [13], try to explain how people are managing information concerning their health. They highlighted that information influences emotional and cognitive representations, which could lead to an adaptive or a maladaptive change of behavior. Genetic notification has an important psychological and emotional impact [14]. In the case of smoking, it could influence the motivation to quit smoking or lead to fear and depression symptoms that depend on the individual, the type of notification and the way it is done. Hence, being one of the core tasks of Public Health Genomics, genetic risk communication is challenging because an individual may interpret the risk as an absolute prediction. For example, he may believe that a high genetic risk of lung cancer will absolutely lead to cancer [15]. However, in general, benefits of genetic tests are more important than risks [4].

Studies reporting the impact of genetic notification on smoking cessation have been conflicting, which could, among other reasons, suggest that it is not a strong motivator of behavioral change.

The impact of genetic notification can be either explored in a real or a hypothetical situation. In hypothetical genetic testing, the anticipated reactions of smokers are assessed in view of a hypothetical genetic risk of smoking-related disease. The outcome of interest is intention-to-quit smoking, which is an important precursor of quit attempts that lead to smoking cessation [16]. Thus improvement in intention-to-quit (e.g. enhanced by genetic notification) could be associated with an increase smoking cessation rate.

The primary objective of this systematic review and pooled-analysis was to determine the impact of genetic notification on smoking cessation in the general population. We addressed this by the following questions:

Is genetic notification of smoking-related disease risk influencing smoking cessation success rate in comparison to clinical notification of smoking-related disease risk (e.g. blood pressure and cardiovascular diseases) or no notification?
Is high genetic risk notification of smoking-related disease risk influencing smoking cessation success rate in comparison to low genetic risk notification?

The secondary objectives were to review the impact of genetic notification on intention-to-quit smoking and emotional outcome as well as to determine to which extend smokers really understand and recall their genetic notification.

## Methods

First, we conducted a systematic review. Then we carried out further quantitative assessment only on the primary outcome (smoking cessation) by a pooled analysis. For this systematic review and pooled-analysis, we followed the Quality of Reporting of Systematic reviews and meta-analyses (PRISMA) guidelines (PRISMA Flow Diagram S1 and PRISMA Checklist S1) [17].

### Eligibility Criteria

Regarding the systematic review, we included studies incorporating smokers of any age receiving genetic notification of smoking-related disease risk in prospective studies (randomized, not randomized trial or cohort studies). The only exclusion criterion was studies involving hypothetical genetic notification as intervention.

The intervention of interest was the genetic notification of smoking-related disease risk. Notification based on one gene was stratified in high and low genetic risk based on dominant or recessive genetic model.

The primary outcome was smoking cessation. To assess the impact of genetic notification, we collected smoking cessation rates at each follow-up that was presented in the studies. Smoking cessation could be biochemically confirmed (salivary cotinine concentration less than 15 ng/ml) or self-reported. The outcomes in the selected studies included prolonged (continuous abstinence during a follow-up period) and point prevalence (1, 7 or 30-day) cessation rates. Only one study presented a sustained smoking cessation [18]. For the pooled-analysis, we focused on point prevalence smoking cessation, as this indicator was available for each study. Velicer et al demonstrated a high correlation between the different types of point prevalence (24-h, 7-day, 30-day) smoking cessation (r between 0.98 and 0.99) [19].

Secondary outcomes were: (a) intention-to-quit smoking; (b) emotional outcome (e.g. anxiety, depression or fear); (c) recall and understanding of the genetic notification.

### Search Strategy

We searched PubMed, Embase, Scopus, Web of Science, PsycINFO, and Toxnet for studies published until August 2011 without language restriction using the following terms: smoking cessation, genetic testing, and genetic predisposition to disease. The search strategy is available in the supporting information documents (PubMed research S1). In addition, we manually reviewed the reference list of relevant articles and reviews.

**Figure 1:**
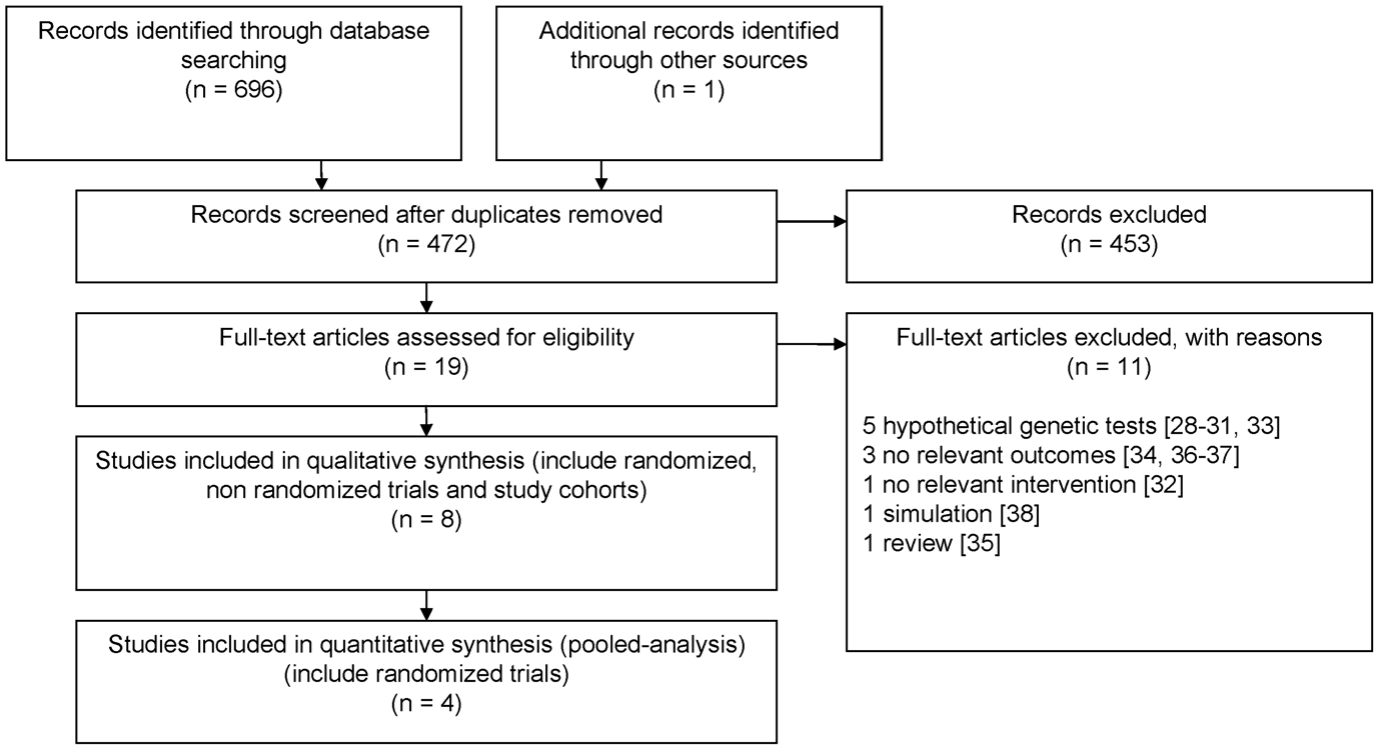
Flow chart of the study selection process.

Two authors (SDV and JVDH) independently screened for title and electronic abstracts identified by the search for relevance to the inclusion criteria. Articles retrieved from this examination were full text screened by the same authors. Reasons for excluding studies were noted (Figure 1). Data were extracted by one author (SDV) and checked by the second one (JVDH). Disagreements were resolved by discussion.

### Data Extraction and Methodological Quality Assessment

From each eligible paper of the systematic review, we extracted information, where available, on first author, year of publication, country, study setting, study design, year of the recruitment, inclusion and exclusion criteria, sample size (total and by comparison groups), description of interventions, characteristics of participants (including age, gender, ethnicity, cigarettes per day, nicotine addiction and, age of initiation), outcomes (smoking cessation, intention-to-quit smoking, emotional effects, recall and understanding of genetic testing and assessment method of the outcomes), and length of follow-up.

Methodological quality and potential risk of bias were assessed using the following criteria: selection criteria clearly described, sample size calculation, adequate allocation concealment for randomized trials, comparability of groups at baseline, presentation of the Hardy-Weinberg Equilibrium (HWE, state that allele and genotype frequency remain constant within a population); intention-to-treat analysis, ascertainment of outcome, and control for confounding.

### Statistical Analyses

In the systematic review, we realized comparisons of sample sizes between studies, genetic risk and gene to understand the difference in the relative proportion of smoking cessation between studies. This was effectuated using Pearson Chi’s square test. Studies included in these analyses were first randomized and non randomized trial [18], [20]–[24]. Sample size of high and low genetic risk was only available in four studies [18], [21], [22], [24].

Individual study risk ratios (RR) and binomial 95% confidence intervals were computed from event numbers extracted from each study.

For the pooled-analysis, we limited included studies to randomized trials for their ability to minimize likelihood of systematic error [25]. Analyses were carried out taking into account time to follow-up: (i) Short-term follow-up was lower or equal to 6 months. (ii) Last follow-up was the last follow-up presented in the study (from 2 to 12 months). We utilized the DerSimonian and Laird method to obtain summary RR and 95% confidence intervals, using random effect models for all analyses because of the important diversity between studies in the inclusion criteria or time of follow-up [26]. Statistical heterogeneity was assessed using the I^2^ statistic, with a value of 50% or more indicating a substantial level of heterogeneity [27]. Potential publication bias was estimated by the Egger’s test. Sensitivity analysis was realized to assess the effect of each single study on the overall results by dropping one study at a time [28].

In studies that had more than two arms, we collapsed arms to obtain one intervention and one control group (e.g. collapsing no intervention and clinical risk notification group). Tests were two-sided with a significance rate α of 0.05. All statistical analyses were performed using STATA, version 10.1 (STATA Corporation Inc., College Station, TX, USA).

## Results

### Description of Studies

The selection of studies included in our review is summarized in Figure 1. The literature search identified 696 publications from the different databases and 1 from hand search [24]. The publication retrieved from hand search was in the reference list of Hishida et al [21]. After removal of duplicate references, 472 were included. A total of 453 were discarded in the title and abstract screening because these papers did not meet the criteria. From the 19 studies included in full text review, 11 did not meet the inclusion criteria as described [29]–[39]. Finally, 8 papers from 7 studies met the inclusion criteria (Table 1) [18], [20]–[24], [40], [41]. The studies of Lerman et al [23] and Audrain et al [20] were based on the same population but presented the outcomes at two and twelve months of follow-up, respectively. Four studies had recruited their participants in 2000 and later [21], [22], [40], [41] and three studies recruited before 2000 [18], [20], [23]. In the last study, the recruitment period was not reported [24]. Studies took place in the UK [24], the US [18], [20], [23] and Japan [21], [22], [40], [41]. Study participants were recruited via newspapers advertisements [20], [23], university [41], annual check-up of employees [21], [40], [41], outpatients consulting general practitioners or specialists [18], [22], smoking clinic [23] or telephone quit smoking service [24]. The sample sizes ranged from 61 to 697 and the follow-up ranged from 2 to 12 months.

**Table 1:** Genetic notification and smoking cessation: Overview of included studies.

Study,Country	Study characteristics	Criteriaon CPD	Intervention/Control group	Genetictest	Samplecharacteristics	Outcomecriteria(method)
Audrain(1997)[20], USA	Recruitment: newspapers,advertisements and smokingclinic; Randomized trial;Last FU: 12 months	≥5 CPD	(a) Standard consultation + COlevel + genetic risk; (b) Standardconsultation; (c) Standardconsultation + CO level	CYP2D6	N = 426; 62.8% female;age range 18 to 75; 83.9%white; FTND mean 5.4;Cont. N.R.	SR (30-dayquitsmoking)
Hamajima(2004)[40], Japan	Recruitment: Annual checkup;Study cohort; Last FU:3 months	N.R.	Genetic risk	GSTM1;GSTT1;NQ01 C609T	N = 101; 31.7% female; agerange 39 to 88; ethnicityN.R.; Add. N.R.; Cont. 89.1%	SR (currentSmokingstatus)
Hishida(2010)[21], Japan	Recruitment: Annual checkupat work place; Non randomizedtrial (Sequentially allocated);Last FU: 12 months	N.R.	(a) Genetic risk; (b) Nointervention	L-*myc*	N = 562; 6.2% female; age20 to >60; ethnicity N.R.;Add. N.R.; Cont. 95.0%	SR (N.R.)
Ito (2006)[22], Japan	Recruitment: First visit outpatientsin Cancer Center; Nonrandomized trial (Sequentiallyallocated); Last FU:9 months (genetic notification:3 month follow-up)	≥1 CPD	(a) Genetic risk; (b) Nointervention	L-*myc*	N = 697; 40.5% female; agerange 20 to 65; Ethnicity N.R.;40.5% FTND from 6 to 10;Cont. 70.0%	SR (currentsmokingstatus)
Kano (2007)[41], Japan	Recruitment: Annual checkupin Municipal government;University (Employees andstudents); Study cohort; LastFU: 3 months	N.R.	Genetic risk	GSTM1;GSTT1;NQ01 C609T;CYP1A1 Ile/Val	N = 107; 14.0% female; agerange 20 to 69; ethnicity N.R.;Add. N.R.; Cont. 68.2%	SR (currentsmokingstatus)
Lerman(1997)[23], USA	Recruitment: newspapers,advertisements and smokingclinic; Randomized trial; LastFU: 2 months	≥5 CPD	(a) Standard consultation + COlevel + genetic risk; (b) Standardconsultation; (c) Standardconsultation + CO level	CYP2D6	N = 427; 61.4% female; agerange 18 to 75; majority ofwhite; CPD mean 22.7;Cont. 60%	SR (7 and30-dayquitsmoking)
McBride(2002)[18], USA	Recruitment: Health clinicfor low income residents(from the adult medicine,dental, urgent care, and specialtyclinic); Randomized trial;Last FU: 12 months	≥1 CPD	(a) Genetic risk; (b) Standardconsultation	GSTM1	N = 557; 60.0% female; agemean 44.5; 100% AfricanAmerican; CPD mean 15.5;Cont. 32%	SR (7-dayquitsmoking)CO level (Salivarysample)
Sanderson(2008)[24], UK	Recruitment: Call on the LondonStop Smoking service the 4previous years; Randomizedtrial; Last FU: 2 months	≥7 cig.in thepast wks	(a) Genetic risk; (b) Nointervention	GSTM1	N = 61; 62% female; agerange 26 to 79; 88% white;CPD mean 19; Cont. N.R.	SR (currentsmokingstatus)

CO level Carbon monoxide level; Cont. Pre-contemplator and contemplator from the stage of behavioral changes of Prochaska *et al* [48]; CPD Cigarette per day; FTND Fageström Test of Nicotine Dependence; FU Follow-up; Add. Nicotine addiction; N.R. Not reported; SR Self-reported abstinence.

Participants were aged from 18 to 88 years old. The percentage of females was around 50% except in two studies where there were only 6.2% and 14.0% females [21], [41]. In general, participants had to smoke at least 1 cigarette per day (CPD) or 7 cigarettes per week to be recruited [18], [22], [24]. Four studies presented the mean CPD of their participants this ranged between 15.5 and 22.7 [18], [20], [23], [24]. Two studies enrolled patients that wanted to quit smoking [20], [23], whereas, in the other studies, participants were not necessarily trying to quit smoking [18], [21], [40], [41].

Regarding the study design, four studies were randomized trials [18], [20], [23], [24], two studies did not randomize their interventions (sequential allocation) [21], [22], and the remaining two were cohort studies [40], [41]. Four studies compared groups that could receive genetic notification, to control [18], [21], [22], [24]. The last two trials had three arms (i) standard quit smoking consultation (QSC) to (ii) clinical risk notification which consisted of 10 minutes of motivational intervention including carbon monoxide (CO) level prior QSC, and (iii) genetic risk notification which consisted in personalized feedback of genetic test, QSC and clinical risk notification [20], [23].

The disease of interest was always cancer. In six studies risk notification was based on a single-gene test (GSTM1, L-myc or CYP2D6) [18], [20]–[24]. In one study three genetic tests (GSTM1, GSTT1 and NQ01 C609T) [40] were involved. In the study of Kano et al CYP1A1 Ile/Val was added to the previous list [41].

### Quality of Studies and Publication Bias

Table S1 presents a summary of the risks of bias for the included studies. One study reported allocation randomization procedures in sufficient details (including the explanation of the procedure for the randomization) [24], whereby intervention allocation were not known or predicted by the participants or the medical support teams before their assignments. The 3 other randomized studies did not mention the procedure of randomization [18], [20], [23]. Studies in which interventions were randomized according to week or month of attendance [21], [22], were considered as non randomized studies in the analyses. Half of the studies reported the sample size calculation [20], [22]–[24] and one clearly reported the test of HWE [21]. Smoking cessation was sometimes defined as a continuous abstinence [22], 7-day abstinence [18], [23], 30-day abstinence [20], [23], or ‘current smoking status’ [21], [22], [24], [40], [41]. Only one study tried to confirm biochemically the self-reported abstinence but the return rate of the samples was only 39%. Thus they decided not to use this outcome and to use only self-reported cessation [18]. Six studies controlled their results for confounding [18], [20]–[24].

Egger’s two tailed p-value showed no significant publication biases for short follow-up as well as the last follow-up of main analyses (genetic notification versus control) (respectively, p = 0.11 and p = 0.76).

### Primary Outcome: Smoking Cessation

**Figure 2:**
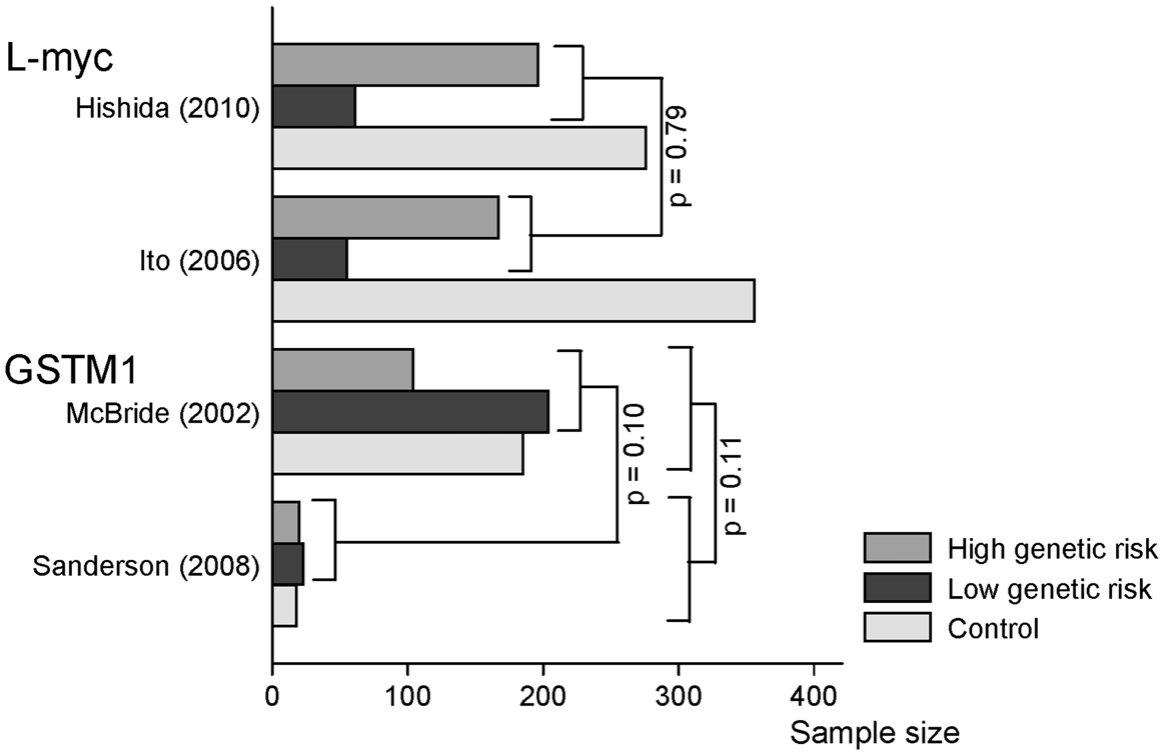
Comparison of the distributions (High, low genetic risk and/or control) between studies or genes. p-values not presented are lower than 0.05.

The impact of genetic notification on smoking cessation was conflicting among randomized and non randomized studies [18], [20]–[24] although the results were not significant. Three studies displayed a higher smoking cessation rate in the genetic notification group than in the control group [18], [23], [24] and three other studies reported the opposite [20]–[22] (Table 2). Figure 2 indicated that, the distribution of high and low genetic risk was significantly different between genes (L-myc and GSTM1; p<0.001) and between the four different studies (p<0.001) but not between authors within genes (GSTM1 p = 0.10; L-myc p = 0.79). For GSTM1, more participants had a lower genetic risk and for L-myc, more participants had a higher genetic risk. However, distribution of high and low genetic risk notification were not available for two studies [20], [23].

**Table 2:** Risk ratios and 95% confidence intervals associated with smoking cessation following intervention (genetic notification versus control).

Author	Gene	Risk Ratio (IC 95%)
Audrain (1997)	CYP2D6	0.73 (0.41; 1.28)
Hishida (2010)	L-myc	0.75 (0.40; 1.41)
Ito (2006)	L-myc	0.90 (0.66; 1.24)
McBride (2002)	GSTM1	1.47 (0.90; 2.39)
Lerman (1997)	CYP2D6	1.44 (0.74, 2.80)
Sanderson (2008)	GSTM1	0.92 (0.37; 2.27)

Risk ratios higher than one mean a positive effect of genetic notification on smoking cessation.

**Figure 3:**
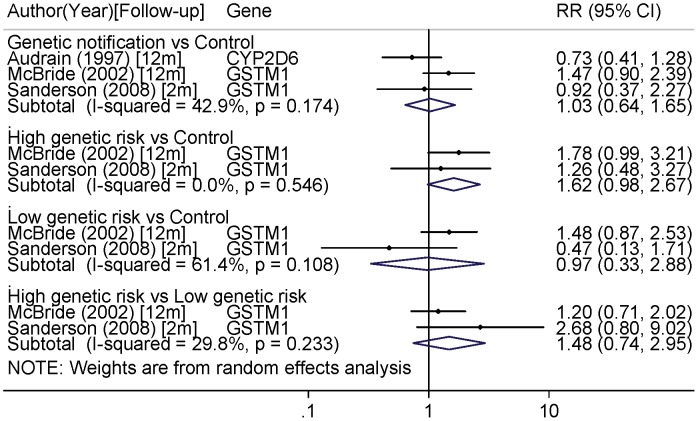
Pooled-analysis of smoking cessation associated with genetic notification in randomized trials for the last follow-up (2, 6 or 12 month). Genetic notification group versus control; High genetic risk versus control; Low genetic risk versus control; High genetic risk versus low genetic risk. Risk ratios higher than one mean a positive effect of genetic notification on smoking cessation.

The pooled analysis indicated that, when considering the last follow-up, there were no significant differences in smoking cessation between the following subgroups: genetic notification versus control, low genetic risk notification versus control, and high genetic risk notification versus low-genetic risk (respectively, RR (95% CI) 1.03 (0.64–1.65); 0.97(0.33–2.88); 1.48(0.74–2.95)) (Figure 3). However, compared to the control group high genetic risk notification was borderline associated with an increased smoking cessation (RR = 1.62 (0.98–2.67)). No heterogeneity was observed across studies in the different pooled-analyses (I^2^ range from 0.0 to 42.9%, p range from 0.17 to 0.54) except a substantial heterogeneity in the low genetic risk notification versus control group (I^2^ = 61.4%, p = 0.11).

**Figure 4:**
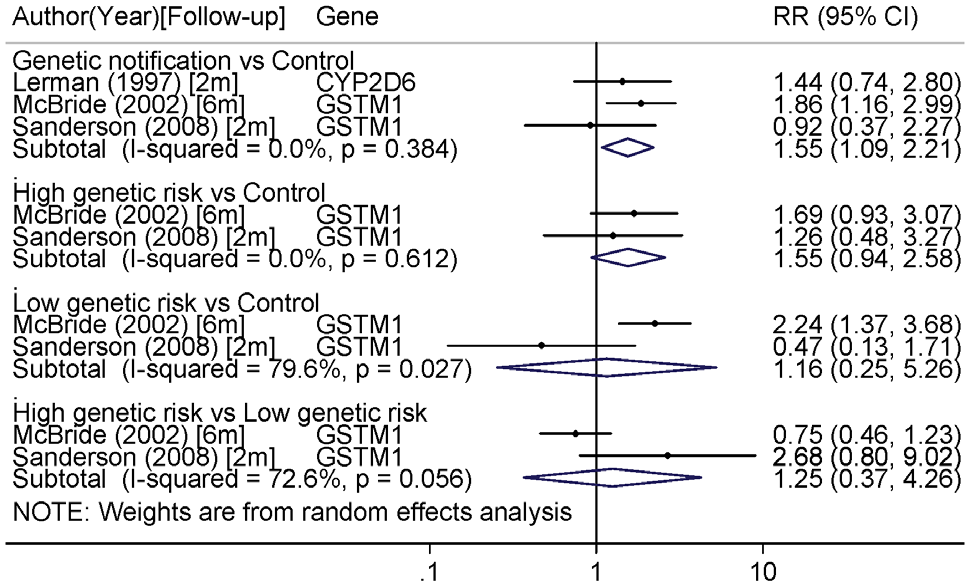
Pooled-analysis of smoking cessation associated with genetic notification in randomized trials for follow-up ≤6 month (2 or 6 month). Genetic notification group versus control; High genetic risk versus control; Low genetic risk versus control; High genetic risk versus low genetic risk. Risk ratios higher than one mean a positive effect of genetic notification on smoking cessation.

When focusing only on short-term smoking cessation outcome (2 or 6 month), the borderline effect of high genetic risk versus control was still visible (RR = 1.55 (0.94–2.58)). Moreover, genetic notification increased 1.55 times smoking cessation in comparison to control (RR = 1.55 (1.09–2.21) (Figure 4). These two analyses seemed fairly homogeneous (I^2^ = 0.0% for the two analyses). Sensitivity analyses did not identify influential studies.

### Secondary Outcomes

Secondary outcomes were available for most of the studies but not all of them.

(a) Intention-to-quit. Six studies observed intention-to-quit smoking after a genetic notification of smoking-related disease risk [18], [21], [22], [24], [40], [41]. However, different notions were used: “wish to quit” [21], [40], [41], “desire to quit” [18] and “motivation to quit” [22], [24]. Most studies just evaluated intention-to-quit smoking at baseline. Four studies presented multiple evaluations over time, such as at baseline, before genetic announcement and three months after announcement; or at baseline, and one week and two months after [21], [24], [40], [41]. Two studies indicated no difference in intention to quit at different time of follow-up [21], [24]. Although, one reported a significant difference in motivation to quit smoking at one week (p = 0.003) but no more at two months [24]. Nevertheless, Ito et al reported no difference of motivation to quit smoking between high and low genetic risk of smoking-related disease (p = 0.18) [22]. In the 6 studies assessing the stage of change, the majority of the population across 5 studies was pre-contemplator or contemplator (no concern or no intention to quit smoking): 89.1% [40], 95.0% [21], 68.2% [41], 60% [23], and 70% [22]. In only one study this percentage was low (32% [18]) (Table 1).

(b) Emotional outcome. Seven studies reported emotional outcome [18], [21]–[24], [41], [42]. Participants receiving genetic notification were more likely to report short-term depression, anxiety or fear arousal than others [21], [23], [24]. More specifically participants with high genetic risk reported increased fear arousal than those with low genetic risk (p<0.01) [22]. This result was not observed in the study of McBride et al [18] and was not significant anymore at 2 months follow-up in the study of Sanderson et al [42]. Anxiety was not significantly different across comparison groups [42].

 (c) Recall and understanding. Smokers seemed to reasonably understand the meaning of their genetic test results [24]. McBride et al reported that half of their participants read the biomarker test result booklet and that an equal number of participants from the high genetic risk and low genetic risk groups interpreted accurately their results (respectively, 56 and 53%) [18].

## Discussion

Our systematic review indicated that few studies have assessed the impact of genetic notification on smoking cessation. Only 8 papers based on 7 different studies were available and just 4 of them were included in the pooled-analysis.

The pooled-analysis suggested a short-term increase of smoking cessation for participants receiving genetic notification in comparison to control group and a borderline increase of smoking cessation for high genetic risk smokers in comparison to control. No evidence of heterogeneity was observed for these results even if (i) characteristics of participants, (ii) inclusion criteria and (iii) study designs differed among studies. For example, (i) included participants were not necessarily interested in smoking cessation. Furthermore, participants were not necessarily from the same ethnicity, although mixing ethnicities in genetic studies could lead to population stratification. The majority of studies did not report HWE calculation. (ii) Inclusion criteria differed in number of CPD and none of the studies met the criterion of nicotine addiction, often defined as smoking more than 10 CPD for at least 12 months [43]–[45]. (iii) Control groups in the study design received either no intervention [18], [24] or clinical risk notification and no intervention [20], [23]. These factors may have affected our results. However, for example for the control groups, collapsing no intervention to clinical risk notification did not introduce a significant change in comparison to no interventions alone for both Audrain et al [20] or Lerman et al [*23*]. Moreover, this did also did not influence significantly the results of the total pooled analysis.

The reason why some results of the pooled-analysis were significant and others were not remains open to interpretation. The impact of genetic notification is likely to vary with individual characteristics (e.g. willingness to quit smoking, socioeconomic status or health literacy), with the distribution of high and low genetic risk notification, with the way in which the genetic notification is done (e.g. counseling before genetic notification, only oral explanation or leaflet with graphical illustration), the intervention in the control group, and the length of the follow-up. Due to the low number of included studies, it was not possible to stratify the pooled-analyses by time of follow-up (2, 6 or 12 months). Stratification of the results before and after 6 months follow-up should be also of interest. However, after 6 months there were only 2 studies available for genetic notification versus control [18], [20] and only one for the stratified analyses (high genetic risk versus control; low genetic risk versus control; high versus low genetic risk) [18]. This enhances the need of increasing the follow-up of studies about the impact of genetic notification on smoking cessation. The inclusion of new studies in the pooled-analysis could improve the power of the analyses. This could either confirm our current results or in the opposite, present a significant impact of genetic notification in long-term follow-up. However, current results do not demonstrate any evidence of long term effect of genetic notification on smoking cessation. And in a population receiving high genetic risk notification, only one study reported a marginally non-significant long-term effect [18].

In a recent meta-analysis the same primary outcome was studied, but there were differences in the method that was used [46]. In our study, we decided to focus on randomized trials only for their ability to minimize the likelihood of systematic errors. Smerecnik et al included both randomized [18], [20], [23], [24] and non randomized trials [21], [22]. In order to avoid overestimation of a single study we considered one follow-up of each study in the pooled analysis (last follow-up or short-term smoking cessation), whereas this was not the case in the other study. We decided to use RR rather than OR for their easiness of interpretation and for their improved accuracy in prospective studies. In our study we also assessed secondary outcomes (intention-to-quit smoking, emotional outcome and recall and understanding of the genetic information). Despite the differences the main outcome of both studies is similar, which reinforces the validity of the results.

Genetic notification did not influence intention-to-quit smoking, except in one study reporting motivation to quit at one week follow-up [24]. In hypothetical genetic tests, higher anticipated intention-to-quit was reported in genetic notification in comparison to the control group [32] and in the high genetic risk group in comparison to the low genetic risk group [29]–[31], [34]. This discrepancy seemed to demonstrate that the anticipated reaction in hypothetical genetic tests did not represent reality. However, this could be due to divergence in the presentation and the understanding of genetic notification or differences in the characteristics of the population. The emotional outcome could also influence this result. In hypothetical genetic tests, smokers will probably be less influenced by depression, anxieties and fear arousal than in real genetic tests. Thus, how smokers recall and understand genetic notification as well as how they are influenced by emotional outcome could improve the use of this intervention in smoking cessation. In the pooled-analysis, most participants were in quite strong intention-to-quit at baseline [18], [24]. However, this variable was not assessed in the 2 last studies [20], [23].

Most studies were testing only one single gene to determine smoking-related disease risk. This posed also ethical questions because of the uncertainty of disease risk which is enhanced when using only single gene test in common diseases.

Genetic notification is one possible intervention among others. At the individual level, the most well-known one are the pharmaceutical interventions (nicotine replacement therapies, Bupropion or Varenicline). However, nicotine dependence is not only a physical dependence but also a behavioral and a psychological dependence. Consequently, interventions might take into account these 3 types of dependence (e.g. multidisciplinary follow-up including psychological counseling and pharmaceutical treatments). Other interventions are also available at the household level (e.g. smoke-free home and partner support) and the society level (e.g. mass media, package warning and bans). As the evidence for benefit of these interventions is strong and well-established, it is incumbent upon genetics to demonstrate additional benefit [47].

### Limitations of the Review

Regarding the pooled-analysis, the most important limitation was the low number of included studies, which did not allow us to determine whether the risk varied with particular conditions (e.g. history of smoking-related disease or stage of behavioral change of Prochaska et al [48]). This low number of included publications was even more present in the pooled-analysis. This is also explained by the fact that we would include only randomized trials that are known to be of higher quality [25]. Publication biases are in general the principal methodological limitation in meta-analyses. It is possible that we missed unpublished reports. The Egger statistical analysis, which is a test for publication biases, suggested that there were no small study effects (p-value comprised between 0.11 and 0.76). However, the sensitivity of this test is generally low in meta-analyses based on fewer than 20 studies [49]. Finally, we did not control our results for multiple testing.

Some limitations pertained to the studies themselves. The outcome measures differed across the studies (e.g. smoking cessation: prolonged abstinence or different point prevalence abstinence). Moreover, adjusted RRs were rarely presented in the included studies, which prevented control for confounding factors. Another limitation was that interventions in the control groups were not similar in the different studies included in the pooled-analysis: two studies had a control group without any intervention [18], [24] and the two others had two control groups (no intervention and clinical risk notification) [20], [23]. The latter have been collapsed in the pooled-analyses, which might dilute the effect of genetic notification on smoking cessation.

Finally, limitations are also due to the heterogeneity between the included studies. This is due to the diversity in the inclusion criteria. For example, the mean number of CPD ranged from 15.5 [18] to 22.7 [20], [23] depending on the study. Nevertheless, to take this problem into consideration we used random effect models in the pooled-analyses.

### Implications for Practice and Research

The results from this study suggest that genetic notification of smoking-related disease risk could have a positive impact on smoking cessation, particularly in short-term follow-up. To determine the possible implications for practice, further research of the impact of genetic notification on smoking cessation is needed. There is also need to investigate the cost-effectiveness of this intervention. Studies should (i) focus on smokers that want to quit smoking, (ii) focus on population of regular smokers by level of severity of nicotine addiction, (iii) use combination of genetic tests for a single or multiple smoking-related diseases, (iv) standardize different concepts (e.g. smoker, addiction, intention-to-quit, smoking cessation) to minimize the heterogeneity and risk of bias between studies.

## Supporting Information

Table S1 **Quality assessment of studies included in the systematic review of genetic notification.** Quality assessment of the studies included in the systematic review and the pooled-analysis.+ Yes; - No or not reported; ? unclear;/Not applicable; ^1^No explanation on how the randomization was processed; ^2^Significant difference in desire to quit at baseline (but not corrected for multiple testing); ^3^Not even try to confirm biochemically the smoking status.
(DOC)

PubMed research S1 ***PubMed research.*** PubMed research used regarding the article selection.
(DOC)

PRISMA Flow Diagram S1 **PRISMA flow diagram.** PRISMA 2009 flow diagram regarding the article selection.
(DOC)

PRISMA Checklist S1 **PRISMA Checklist.** PRISMA 2009 checklist regarding the pooled-analysis data and position in the manuscript.
(DOC)
