# Supplementary material for: Impact of Genetic Notification on Smoking Cessation: Systematic Review and Pooled-Analysis
Source: PLoS One. 2012 Jul 11;7(7):e40230. doi: 10.1371/journal.pone.0040230 (PMC3394798; doi:10.1371/journal.pone.0040230)
Supplement: Table S1 — Quality assessment of studies included in the systematic review of genetic notification. Quality assessment of the studies included in the systematic review and the pooled-analysis.+ Yes; - No or not reported; ? unclear;/Not applicable; 1No explanation on how the randomization was processed; 2Significant difference in desire to quit at baseline (but not corrected for multiple testing); 3Not even try to confirm biochemically the smoking status. (DOC) [file pone.0040230.s001.doc]

## Supporting information table

Table S1. Quality assessment of studies included in the systematic review of genetic notification

|  | **Selection criteria clearly described** | **Sample size calculation** | **Adequate allocation concealment** | **Comparability of groups at baseline** | **Presentation of the HWE** | **Intention-to-treat analysis** | **Ascertainment of outcome** | **Control for confounding** |
| --- | --- | --- | --- | --- | --- | --- | --- | --- |
| **Studies included in the systematic review and pooled analysis** | | | | | | | | |
| Audrain 1997 | + | + | +/-^1^ | + | - | - | -^3^ | + |
| Lerman 1997 | + | + | +/-^1^ | + | - | - | -^3^ | + |
| McBride 2002 | + | - | +/-^1^ | +/-^2^ | - | + | + | + |
| Sanderson 2008 | + | + | + | + | - | + | -^3^ | + |
| **Studies just included in the systematic review** | | | | | | | | |
| Hamajima 2004 | - | - | / | / | ? | - | -^3^ | - |
| Hishida 2010 | - | - | - | + | + | - | -^3^ | + |
| Ito 2006 | +/- | + | - | + | - | + | -^3^ | + |
| Kano 2007 | - | - | / | / | - | + | -^3^ | - |

+ Yes; - No or not reported; ? unclear; / Not applicable;

^1^ No explanation on how the randomization was processed;

^2^ Significant difference in desire to quit at baseline (but not corrected for multiple testing);

^3^ Not even try to confirm biochemically the smoking status.
