# Supplementary material for: Impact of Genetic Notification on Smoking Cessation: Systematic Review and Pooled-Analysis
Source: PLoS One. 2012 Jul 11;7(7):e40230. doi: 10.1371/journal.pone.0040230 (PMC3394798; doi:10.1371/journal.pone.0040230)
Supplement: PubMed research S1 — PubMed research. PubMed research used regarding the article selection. (DOC) [file pone.0040230.s002.doc]

## Pubmed research S1

The research on Pubmed was realized using the following code:

((smoking cessation) AND ((genetic testing) OR genetic predisposition to disease)))
