# Supplementary material for: Impact of Genetic Notification on Smoking Cessation: Systematic Review and Pooled-Analysis
Source: PLoS One. 2012 Jul 11;7(7):e40230. doi: 10.1371/journal.pone.0040230 (PMC3394798; doi:10.1371/journal.pone.0040230)
Supplement: PRISMA Flow Diagram S1 — PRISMA flow diagram. PRISMA 2009 flow diagram regarding the article selection. (DOC) [file pone.0040230.s003.doc]

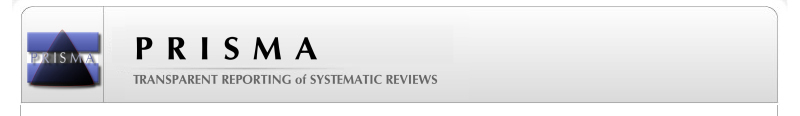
**PRISMA 2009 Flow Diagram**

**Screening**

**Included**

**Eligibility**

**Identification**

Records identified through database searching
(n = 696)

Additional records identified through other sources
(n = 1 )

Records after duplicates removed
(n = 472)

Records screened
(n = 472 )

Records excluded
(n = 453 )

Full-text articles assessed for eligibility
(n = 19 )

Full-text articles excluded, with reasons
(n = 11 )

Studies included in qualitative synthesis
(n = 8 )

Studies included in quantitative synthesis (meta-analysis)
(n = 4 )
